# 

**Published:** 1916-10

**Authors:** 


### Publishers' Announcement

The idea among some folks that advertising is only of service to the seller, is incorrect. If advertising were only of service to the advertiser, advertising would have been dead long ago, and some other means of informing the buying public what was at their disposal for a certain price, would be in vogue. This would be advertising too. So advertising is a living thing and may be said to have a thousand lives, for if one form would become obsolete, it would spring to life again in some other form and take on new life. Thus it is that advertising is up-to-date and forever reverberating around the popularly demanded merchandise.

The buyer knows thoroughly what is on the market by watching the advertisements and can not be eclipsed by an unknown article or a "dark horse." An unknown article never is heard of in advertising and unless advertised, remains unknown. Unless an article is good enough to stand advertising, it can not be advertised because advertising is the spot light of publicity and brings out defects as easily as merits of merchandise.

Advertised brands are safe for the public to buy because they stand the test of publicity.

### ANNOUNCEMENTS

OFFICERS NATIONAL DENTAL ASSOCIATION. President, Dr. L. L. Barber, Toledo O.; President Elect, Dr. W. H. G. Logan, Chicago, Ill.; First Vice President, Dr. A. P. Burkhart, Auburn, N. Y.; Second Vice President, Dr. M. E. Vance, Lincoln, Neb.; Third Vice President, Dr. H. C. Hassell, Tuscaloosa, Ala.; Secretary, Dr. Otto U. King, Huntington, Ind.; Treasurer, Dr. A. R. Melendy, Knoxville, Tenn.; Trustees, Drs. W. E. Boardman, Boston, Mass.; C. J. Grieves, Baltimore, Md.; M. L. Ward, Ann Arbor, Mich.

The next meeting will be held in New York City, probably in November.

The annual dues, payable through the State Societies, will hereafter be two dollars instead of one. It is planned to use the increased income for publishing the Journal monthly hereafter instead of quarterly.

---

PREPAREDNESS LEAGUES OF AMERICAN DENTISTS. Through the recent action of the National Dental Association at Louisville, the Preparedness League of American Dentists may now be considered an affiliated body, co-operating with it through an advisory committee of five of its members empowered to act with the present officers and trustees of the League. The Journal of the National Dental Association is to be its official organ, through which League members will be regularly informed of all matters pertaining to this great work.

It is the object and desire of the League to aid the War and Navy Departments in every possible way, and it is requested by a representative of the War Department that the League secure, as soon as possible, a list of all oral surgeons who will serve in time of war. Those who desire may, after a satisfactory examination, join the Officers'

Reserve Dental Corps with the commission of First Lieutenant.

Units are being formed for the study of the surgery of gunshot wounds about the jaws and face as well as other injuries incident to war which must be treated by the dentist. Those interested in having a unit formed in their locality should so indicate to the League, and immediately steps will be taken to supply the need.

A statement accompanying replies as to experience, college and date of graduation, whether member of National Dental Association or other societies, would be of great assistance in arranging the list. Address The Preparedness League of American Dentists, 3 Professional Bldg., 131 Allen St., Buffalo, N. Y.

---

SIXTIETH ANNIVERSARY MEETING ST. LOUIS DENTAL SOCIETY. The St. Louis Dental Society invites the profession to meet with them November 16, 17, 18, 1916, to celebrate the sixtieth anniversary of the society's organization.

An unusual program of varied interest is being prepared for the educational feature coincident with the spirit of commemoration and good fellowship which will prevail.

A report of the recent experiments and proposed investigations of the Research Institute of the National Dental Association, particularly in relation to systemic manifestations of oral infection, efficiency of ionization, and practicability of various root canal filling materials will be given with motion picture illustrations.

A resume of the present radical tendencies in operative dentistry with a conservative course of procedure will be presented by an eminent authority on the subject. Some timely counsel from one whose opinion commands respect.

The complete technic of aseptic root canal operations will be illustrated and described by one of the well known pioneers in this field. Probably no other class of dental operations is being criticised as this, and its requirements are much more exacting than in the past.

A method of utilizing teeth with vital pulps for bridge abutments will be taught by a constructive genius whose technic is an inspiration. This offers a partial solution to the root canal problem.

Scientific construction of artificial dentures with special attention to partial dentures will be discussed by a prosthesist noted for his ability to teach.

Two afternoons will be devoted to the clinical program, all technic of modern practice being on display by men chosen for their proficiency in special phases. A distinctive innovation will be a series of clinical symposiums in which each step of the operation is detailed instead of the finished product being shown with a description of the process.

This is to be a meeting worth any reasonable effort to attend, a composite and the step in advance of all the "post graduate," "cradle of dentistry," "preparedness," "feel at home" meetings of the past.

If you are dead lie down; if not, line up with the live ones. November 16, 17, 18.

---

OHIO STATE DENTAL SOCIETY. The fifty-first annual meeting of the Society will be held in Dayton, Ohio, December 5, 6, 7, 1916. The complete program will be published in the November *Register.*

---

MICHIGAN SATE BOARD OF DENTAL EXAMINERS. The next regular meeting of the Michigan State Board of Dental Examiners for the examination of applicants who may desire to practice dentistry in Michigan, will be held in the Dental College at Ann Arbor, beginning Monday, November 13, 1916, at eight o'clock a. m., and will continue through Saturday, November 18th. All applications and credentials, together with fees *must be in the hands* of the secretary at least ten days prior to the date of examination. For application blanks and all further information, application should be made to, E. O. Gillespie, Secy.-Treas., Stephenson, Mich.

---

ILLINOIS STATE BOARD OF EXAMINERS will meet in Chicago, at the Chicago Dental College, November 13, 1916, at nine a. m. Information can be had of the Secretary, Dr. O. H. Seifert, Springfield, Ill.

